# A Case of Pulmonary Vein Thrombosis Associated With Treatment of Omalizumab

**DOI:** 10.1177/2324709617724176

**Published:** 2017-08-04

**Authors:** Sandhya Narukonda, Nagadarshini Ramagiri Vinod, Medha Joshi

**Affiliations:** 1 Conemaugh Memorial Medical Center, Johnstown, PA, USA

**Keywords:** pulmonary vein thrombosis, omalizumab, severe persistent asthma, arterial thrombotic events

## Abstract

Pulmonary vein thrombosis (PVT) is a challenging diagnosis and has been described in association with or as a complication of pulmonary tumors, lung surgeries, atrial myxoma, and after radiofrequency catheter ablation for atrial fibrillation. There are not many reported cases of PVT associated with medication use. We present a case of a 53-year-old male with a history of severe persistent asthma on omalizumab, who presented with shortness of breath and was found to have PVT on computed tomography scan of the chest. The hypercoagulable workup was normal, and the patient did not have a history of malignancy or pulmonary surgeries. Currently, available data suggest an association between omalizumab use and increased risk of arterial thrombotic events. However, on a literature search, we could not find any reported cases of PVT with omalizumab treatment.

## Introduction

Pulmonary vein thrombosis (PVT) is a rare entity, which has been a reported complication of pulmonary malignancy, lung transplantation, pulmonary lobectomy, and radiofrequency ablation for atrial fibrillation.^1,2^ The diagnosis is challenging secondary to nonspecific clinical presentations with dyspnea, cough, pleuritic chest pain, and hemoptysis. We present a case of PVT in a patient on treatment with omalizumab for severe persistent asthma.

## Case Presentation

**Figure 1.**
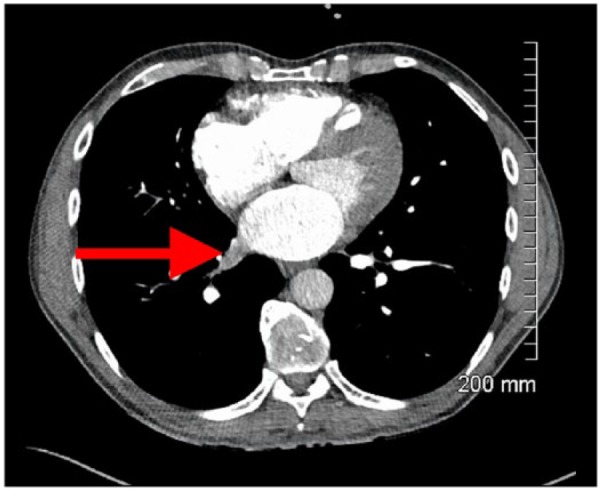
Red arrow shows pulmonary vein thrombosis.

**Figure 2.**
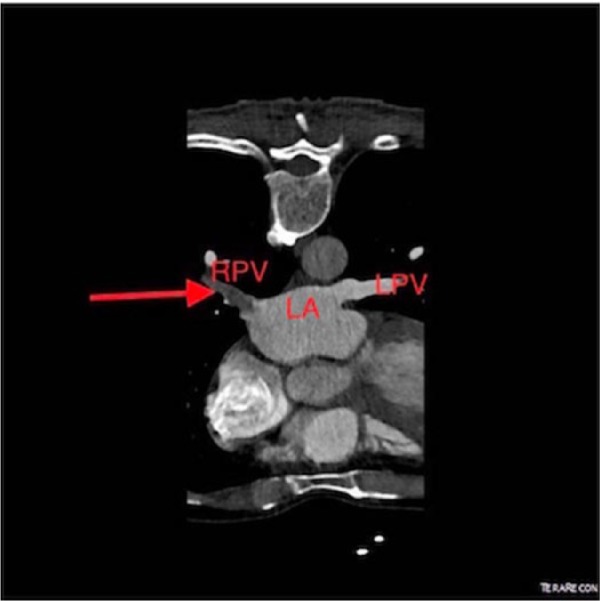
Computed tomography chest with pulmonary embolism protocol. Red arrow shows pulmonary vein thrombosis. RPV, right pulmonary vein; LPV, left pulmonary vein; LA, left atrium.

A 53-year-old Caucasian male presented to the emergency department with complaints of shortness of breath, wheezing, and cough. He had past medical history significant for multiple sclerosis, severe persistent asthma, and allergies. As conventional treatment for asthma could not control the symptoms, the patient was started on omalizumab. The patient was being treated with omalizumab for 2 years for underlying allergic asthma. He had no recent surgical interventions on the lungs, no family history of hereditary thrombophilia, and no history of atrial fibrillation. On admission, the vital signs were normal, and physical examination was unremarkable except for diffuse wheezing on lung auscultation. A chest computed tomography (CT) with pulmonary embolism protocol revealed thrombosis of the right inferior pulmonary vein and thrombosis extending to but not into the left atrium (as shown in Figures 1 and 2). This was a new finding compared to a chest CT obtained 3 years ago. There was no evidence of intrapulmonary neoplasm. The patient was started on intravenous heparin based on the CT evidence of PVT. An echocardiogram was obtained, which showed a right ventricular ejection fraction of 55%, grade I diastolic dysfunction, no intracardiac thrombus, and mild pulmonary vascular hypertension. A hypercoagulable workup was negative for ANA, ANCA, anti-cardiolipin, anti-phospholipid antibodies, prothrombin gene mutation, and factor V Leiden. We suspected that omalizumab might have contributed toward the development of PVT and was discontinued. The patient’s anticoagulant treatment was transitioned from heparin to rivaroxaban. Once the patient’s symptoms improved he was discharged home.

## Discussion

The incidence of PVT is unclear secondary to its rare presentation.^3^ The usual presenting symptoms are cough, dyspnea, pleuritic chest pain, and hemoptysis, which are nonspecific. It might present after pulmonary lobectomy, lung transplantation, lung tumors, radiofrequency ablation for atrial fibrillation, and sclerosing mediastinitis.^1,2^ The postulated mechanisms for the pathogenesis are epithelial damage from surgery or tumor invasion, direct tumor extension into the vein or vein compression from the tumor, pulmonary venous stasis from pulmonary fibrosis, and hypercoagulable states in nephritic or antiphospholipid syndrome.^4,5^ However, sometimes PVT can present without an obvious cause. If not treated in a timely manner, it can lead to life-threatening complications like pulmonary gangrene and peripheral embolization.^4^ Diagnostic modalities used for the diagnosis include transthoracic echocardiography, transesophageal echocardiography, ventilation perfusion scanning, pulmonary angiogram, CT angiogram, and magnetic resonance imaging. Magnetic resonance imaging is useful in differentiating bland thrombus from a thrombus due to malignancy.^4^ Once the diagnosis is made the treatment includes anticoagulation, antibiotic therapy based on the underlying cause, and thromboembolectomy and pulmonary lobectomy used in life-threatening scenarios. Anticoagulation plays an important role in recanalization of the pulmonary vein and preventing the development of a clot in the left atrium.

Omalizumab is anti-IgE monoclonal antibody that has been approved by the Food and Drug Administration since 2003 for the treatment of moderate to severe persistent asthma whose symptoms are not adequately controlled with conventional treatments. Omalizumab has shown to decrease the rate of asthma exacerbations and use of inhaled corticosteroids.^6,7^ Studies have shown that the arterial thrombotic incidents including stroke, cardiovascular mortality, acute coronary syndrome, angina, ischemic heart disease, myocardial infarction, and thrombosis are higher in patients treated with omalizumab.^8,9^ There are no postulated mechanisms available for omalizumab as a cause of thrombosis. The EXCELS study (Evaluating Clinical Effectiveness and Long-Term Safety in patients with Moderate-to-Severe Asthma) revealed that there is an increase in the risk of arterial thrombotic events in patient groups exposed to omalizumab compared with the unexposed group.^10^ However, treatment with omalizumab did not show an increased risk of malignancy.^11^

Our patient presented with symptoms of shortness of breath, wheezing, and cough along with a diagnosis of PVT on CT angiogram. We could not find any other identifiable cause for PVT other than treatment with omalizumab. As omalizumab is associated with increased risk of arterial thrombotic events, given the absence of any other identifiable causes of PVT in our patient, we could attribute omalizumab in the occurrence of PVT. To our knowledge, this was the first reported case of PVT from omalizumab.

## Conclusion

As PVT is a rare diagnosis with a vague clinical presentation, a high index of suspicion is required for early diagnosis and treatment. Delay in diagnosis can be life threatening. Though treatment with omalizumab is promising with respect to improved symptoms in patients with severe persistent asthma who failed conservative treatment, PVT should be considered as an adverse effect with long-term omalizumab use. No case reports are available in the literature showing its association with omalizumab use. To further strengthen the association or causation of omalizumab to thrombotic events, future cohort studies on patients receiving omalizumab treatment are needed.
